# Executive Function Profiles in ADHD and Dyslexia: A Mixed-Method Neurocognitive Analysis

**DOI:** 10.3390/neurolint18060110

**Published:** 2026-06-03

**Authors:** Geanina Cucu Ciuhan

**Affiliations:** Department of Psychology, Communication Sciences and Social Work, Faculty of Educational Sciences, University Center Pitesti, National University of Science and Technology POLITEHNICA Bucharest, Targu din Vale Street no. 1, 110040 Pitesti, Romania; geanina.ciuhan@upb.ro

**Keywords:** executive functioning, ADHD, dyslexia, neurodevelopmental disorders, neurocognitive profiles, mixed-methods

## Abstract

**Background/Objectives**: Executive function (EF) impairments are common in neurodevelopmental disorders but are often examined using group-level approaches that may overlook clinically meaningful cognitive heterogeneity. This study explored EF heterogeneity in children with attention deficit hyperactivity disorder (ADHD), developmental dyslexia, and comorbid presentations using a clinically grounded mixed-method approach. **Methods**: Standardized neuropsychological data from the NEPSY-II, WISC-IV, and Woodcock–Johnson IV batteries were integrated with a case-based thematic synthesis of 11 clinical evaluations. Semi-inductive analysis was informed by preliminary patterns observed in a larger clinical sample. **Results**: Three executive function profiles were identified: (1) globally reduced executive functioning, characterized by widespread deficits in inhibition, attention, and working memory; (2) verbal–mnestic executive vulnerability, marked by weaknesses in verbal memory and attention regulation despite relative cognitive strengths; and (3) selective executive control deficit, reflecting impairments in inhibitory control and self-regulation. These profiles revealed clinically meaningful patterns that were not fully captured by categorical diagnostic classifications. **Conclusions**: The findings support the value of integrated, profile-based approaches for understanding executive function heterogeneity in neurodevelopmental conditions. Such approaches may enhance ecological validity in assessment and contribute to individualized intervention planning. Given the exploratory and case-based nature of the study, the findings should be considered preliminary and hypothesis-generating.

## 1. Introduction

Executive functions (EFs) refer to a set of higher-order cognitive processes—including inhibition, working memory, and cognitive flexibility—that are essential for behavior regulation, attention control, and goal-directed problem-solving [1]. These functions play a critical role in learning, emotional regulation, and adaptive functioning throughout childhood.

Deficits in EF are commonly observed in neurodevelopmental disorders, particularly attention deficit hyperactivity disorder (ADHD) and developmental dyslexia. Attention Deficit Hyperactivity Disorder (ADHD) is currently conceptualized in three clinical presentations—predominantly inattentive, predominantly hyperactive/impulsive, and combined—each associated with partially distinct executive-function profiles [2]. Children with the predominantly inattentive presentation typically exhibit difficulties in sustained attention, working memory, and processing speed, often manifesting as disorganization, forgetfulness, and reduced task persistence [3,4]. In contrast, the hyperactive/impulsive presentation is more strongly linked to deficits in inhibitory control, response regulation, and delay aversion, leading to impulsive behaviors and difficulties in behavioral self-monitoring [4,5]. The combined presentation reflects a convergence of these difficulties, with impairments across multiple executive domains, including attention regulation, inhibition, and cognitive flexibility [3]. However, growing evidence suggests that executive function deficits are neither universal nor specific to any single ADHD presentation, and substantial intra-group variability exists [4,6]. This heterogeneity underscores the importance of moving beyond categorical subtype distinctions toward more nuanced, profile-based approaches that capture individual patterns of cognitive strengths and vulnerabilities.

ADHD is typically associated with impairments in inhibition, working memory, and attentional control [3], while dyslexia is primarily linked to deficits in phonological processing and verbal working memory [7]. These impairments affect not only academic achievement but also social–emotional adaptation, underscoring the importance of individualized cognitive assessments. Importantly, comorbidity between ADHD and dyslexia is frequent, with reported overlap ranging from 25% to 40% [8]. In such cases, EF difficulties may compound, yielding complex and heterogeneous cognitive presentations that challenge standard diagnostic approaches.

The study of comorbid ADHD and dyslexia is particularly important because co-occurring neurodevelopmental conditions may produce additive or interactive executive-function impairments that differ qualitatively from those observed in either disorder alone. Children with comorbid presentations frequently exhibit broader difficulties in inhibition, working memory, processing speed, and academic self-regulation, contributing to more severe functional impairment and more complex intervention needs [8,9]. Understanding how executive function patterns manifest across comorbid presentations may therefore support more precise neuropsychological characterization and more individualized educational and clinical intervention planning.

Despite progress, most EF research in neurodevelopmental disorders relies on variable-centered, group-level analyses that may overlook meaningful intra-individual variation. This limits their relevance for clinical decision-making, which requires integration of multi-domain test results in contextually grounded ways. Few studies have leveraged real-world assessment data to build typological cognitive profiles that reflect functional diversity. Recent person-centered studies using latent profile, cluster, and network analyses have identified distinct executive-function profiles in children and adolescents with ADHD, often based on large samples and multidimensional assessment batteries [10,11,12]. For example, Shan-hong Zhang and colleagues demonstrated that cluster analysis integrating executive function and psychopathological domains can identify clinically meaningful ADHD subgroups with distinct functional impairments, supporting dimensional and transdiagnostic approaches to neurodevelopmental heterogeneity.

This approach is conceptually aligned with the Research Domain Criteria (RDoC) framework, which emphasizes dimensional, transdiagnostic constructs—particularly within the domain of cognitive control—across traditional diagnostic boundaries.

Qualitative synthesis techniques, particularly those based on case-based and semi-inductive approaches, offer promising avenues for capturing heterogeneity in EF across clinical populations [13,14]. Mixed-method designs that combine descriptive statistics with thematic interpretation of test data can support more clinically relevant insights than group means alone—especially in small samples where statistical significance may be unattainable but clinically important trends are present.

Emerging evidence suggests that EF difficulties can be detected early and may follow diverse developmental trajectories, shaped by both neurobiological and environmental influences [15,16]. Typological profiling of EF may provide a bridge between standardized assessment and functional formulations, helping to tailor intervention strategies across home, school, and therapy settings.

Moreover, research on neurocognitive comorbidities in ADHD and dyslexia emphasizes the presence of additive and overlapping deficits. For instance, Moura et al. [15,16] reported that children with both ADHD and dyslexia exhibited weaker performance across domains such as working memory, processing speed, and phonological processing—highlighting the complexity and severity of comorbid presentations.

The multidimensionality of executive functioning is further supported by latent variable research. Furey et al. [6] found that the Delis–Kaplan Executive Function System (D-KEFS) reflects interrelated but distinct executive domains—namely inhibition, shifting, and fluency—overlaid by a general executive factor. These findings support the interpretation of clustered subtest patterns in clinical assessments and align with the case-based profiling approach adopted in the present study.

More recently, Cumming et al. [15] identified latent executive function profiles in young children at risk for emotional and behavioral disorders, reinforcing the relevance of EF profiling in early identification and support. These person-centered approaches further underscore the need to move beyond traditional diagnostic groupings to identify functional cognitive patterns with clinical significance.

Similarly, Becker et al. [10] demonstrated that EF profiles in adolescents with ADHD were more predictive of functional outcomes than symptom categories alone. Such findings provide robust support for clinically driven executive profiling and suggest practical implications for tailoring educational and therapeutic interventions.

The present study responds to this need by adopting a case-based thematic synthesis approach to explore executive function diversity among children with ADHD, dyslexia, or comorbid presentations. By integrating standardized scores from NEPSY-II, WISC-IV, and WJ-IV batteries, we aimed to construct typological profiles that reflect meaningful patterns in EF performance and inform individualized assessment and intervention planning.

Our analysis was conducted in two stages. In Stage 1, we explored descriptive patterns in NEPSY-II executive subtests across a larger clinically assessed sample (N = 40) to identify emerging trends. In Stage 2, we conducted a qualitative case-based thematic synthesis of 11 in-depth clinical evaluations, generating three distinct cognitive-executive profiles through thematic analysis. Together, these findings offer a framework for understanding EF variability in real-world clinical contexts, with implications for diagnostic clarification, intervention targeting, and educational planning.

While recent person-centered research has employed statistical techniques such as latent profile analysis (LPA) to identify subgroups based on executive function indicators [10,11], such approaches typically rely on large datasets and predominantly quantitative modeling. In contrast, clinically grounded contexts often involve smaller samples but richer, multi-informant assessment data that require integrative interpretation.

The present study adopts a mixed-method, case-based approach that combines standardized neuropsychological assessment data (NEPSY-II, WISC-IV, and Woodcock–Johnson IV) with thematic synthesis to explore executive function heterogeneity in children with ADHD, developmental dyslexia, and comorbid presentations. Unlike large-scale latent profile approaches optimized for statistical subgroup detection, the present study emphasizes ecologically grounded clinical interpretation by integrating neuropsychological test performance, academic functioning, and individualized case-level contextualization within a real-world assessment framework.

Preliminary descriptive trends observed in a larger clinical sample informed an in-depth qualitative analysis of individual profiles. The primary aim was to derive clinically meaningful executive function profiles that capture intra-individual variability and cut across diagnostic categories. By identifying distinct cognitive executive configurations, this study seeks to contribute to a more ecologically valid and clinically applicable framework for assessment and individualized intervention planning in neurodevelopmental disorders.

**The current study**

This study adopts a case-based qualitative approach to analyze executive functioning in a clinical sample of children with ADHD, dyslexia, or both. By integrating standardized data from NEPSY-II, WISC-IV, and WJ-IV assessments, we aim to develop typological profiles that capture the heterogeneity of executive functioning in these populations. The goal is to identify clinically relevant executive profiles that can inform diagnosis and individualized intervention planning.

## 2. Methods

### 2.1. Design

This study employed a two-stage, mixed-method design integrating quantitative group-level analysis and qualitative thematic synthesis. In Stage 1, cognitive profiles were compared across diagnostic categories in a larger sample of children assessed using the NEPSY-II battery.

Stage 2 employed a qualitative thematic synthesis design based on 11 individual clinical case reports purposively selected from the larger Stage 1 clinical dataset. The cases were selected from psychological assessments conducted in a private clinical setting, and all met diagnostic criteria for ADHD, dyslexia, or a comorbid presentation of both conditions. The primary aim was to identify recurrent patterns of executive functioning and develop typological profiles applicable in clinical practice. The analytic strategy followed the principles of thematic analysis as outlined by Braun et al. [17], allowing both deductive and inductive identification of pattern constellations across cases.

### 2.2. Participants

The Stage 1 sample initially consisted of 40 children (22 boys, 18 girls) aged 8 to 15 years who had been evaluated in a clinical context using the NEPSY-II. Of these, 30 children met inclusion criteria for group comparison analyses and were classified into three diagnostic categories: ADHD (*n* = 18), developmental dyslexia (*n* = 4), and comorbid ADHD and dyslexia (*n* = 8). Ten additional cases were excluded from group-level analyses due to unclear or mixed diagnostic presentations. These cases were retained in the broader clinical dataset but were not included in statistical comparisons to preserve interpretability.

Where available, ADHD presentations were classified as predominantly inattentive, hyperactive/impulsive, or combined. Information regarding current medication status and comorbid conditions (e.g., ASD) was extracted from clinical records when documented; however, these variables were not systematically available for all participants and were therefore not included in the quantitative analyses.

The Stage 2 sample consisted of 11 children (6 girls, 5 boys), aged between 9 and 13 years (M = 11.0 years), purposively selected from the broader Stage 1 clinical dataset based on the availability of complete multi-instrument assessments (NEPSY-II, WISC-IV, and WJ-IV) and sufficiently detailed clinical reports to support in-depth qualitative analysis. Thus, the Stage 2 sample represented a subsample of the larger clinically assessed group included in Stage 1.

#### Case Selection Strategy (Stage 2)

Cases included in Stage 2 were selected purposively from the larger clinical dataset to represent variability in executive functioning profiles across diagnostic categories. Selection criteria included: (a) availability of complete standardized assessment data (NEPSY-II, WISC-IV, WJ-IV), (b) clear diagnostic formulation based on clinical criteria, and (c) sufficient detail in psychological reports to support cross-case qualitative comparison.

This purposive sampling approach was adopted to enable in-depth, case-based analysis rather than statistical generalization, consistent with qualitative research principles.

### 2.3. Measures

Each child was assessed using a standardized battery of instruments commonly used in clinical neuropsychological evaluation. The Romanian standardized versions of all instruments were employed, and all assessments were conducted by the author, a licensed clinical psychologist with extensive experience in child assessment.

The NEPSY–II [18] is a comprehensive tool for assessing neuropsychological development in children. In this study, selected subtests focused on executive functions, attention, memory, language, and visuomotor integration (e.g., Inhibition, Auditory Attention, Narrative Memory, Design Copying). The Romanian adaptation provided age-appropriate normative data for interpretation.

The Wechsler Intelligence Scale for Children—Fourth Edition (WISC-IV) [19] was used to assess general intellectual functioning. Index scores from the Verbal Comprehension, Perceptual Reasoning, Working Memory, and Processing Speed scales were considered. The Romanian edition ensured cultural and linguistic relevance.

The Woodcock–Johnson IV Tests of Achievement—Form A (WJ-IV ACH A) [20] were used to evaluate academic achievement, with a focus on reading, writing, and phonological processing. Scores were interpreted using age-equivalent metrics (in years and months) to facilitate developmental comparison. The Romanian standardized version was used in all cases.

These instruments were selected for their strong psychometric properties and their ability to capture the executive, cognitive, and academic domains relevant to clinical profiling in children with ADHD, dyslexia, or comorbid presentations.

### 2.4. Procedure

In Stage 1, NEPSY-II subtest scores were extracted from the clinical database and grouped by diagnostic category. Group means and standard deviations were computed across executive function subtests. One-way ANOVAs were conducted to examine differences between ADHD, dyslexia, and comorbid groups. Although statistically significant differences were observed in selected executive function measures, these findings were interpreted cautiously due to the small and uneven group sizes and the exploratory nature of the analysis.

In Stage 2, eleven clinical psychological evaluation reports were selected for inclusion, each documenting the assessment of a child aged 9 to 13 years diagnosed with ADHD (inattentive type), dyslexia, or both. Reports originated from a private clinical practice and were anonymized and labeled as C1 through C11. Each child had been evaluated using the NEPSY-II, WISC-IV, and WJ-IV test batteries, focusing specifically on domains relevant to executive function and academic performance.

Relevant scores were extracted manually and compiled into a structured dataset. For consistency and comparative analysis, Woodcock–Johnson IV achievement scores were transformed into age-equivalent units (years–months). Executive function variables were standardized by developmental norms and classified into three qualitative categories: Low (standard score < 8), Medium (8–11), and High (>11).

Using this coding scheme, a cross-case matrix was constructed to systematically organize executive function indicators across individuals and domains. Performance levels (low, medium, high) were compared across cases to identify recurring patterns of strengths and weaknesses.

Profile identification proceeded through iterative comparison of these matrices, focusing on consistent constellations of executive function performance across domains (e.g., inhibition, attention, working memory). Initial groupings were generated through visual inspection of pattern similarities and were subsequently refined through repeated review of individual case profiles.

To enhance analytic rigor, the classification process was conducted in two iterative cycles. In the first cycle, preliminary profiles were defined based on observed pattern clusters. In the second cycle, all cases were re-evaluated against these definitions, allowing for the refinement of profile boundaries and reclassification of borderline cases where necessary.

This iterative and comparative approach aimed to increase internal coherence and reduce subjectivity in profile assignment, while remaining consistent with qualitative principles of thematic pattern identification.

### 2.5. Rigor and Validity

The analysis adhered to qualitative research standards, including triangulation across instruments, systematic coding procedures, and theory-driven interpretation. Patterns were not generalized beyond the sample but intended to inform hypothesis generation and clinical typologies.

## 3. Results

### 3.1. Stage 1: Descriptive and Comparative Analysis of NEPSY-II Profiles

In Stage 1, data from 30 children with valid NEPSY-II results were included in the comparative analysis across diagnostic categories: ADHD (*n* = 18), dyslexia (*n* = 4), and comorbid ADHD and dyslexia (*n* = 8). Ten additional cases were excluded due to alternative or unclear diagnoses.

Group means and standard deviations were calculated for key NEPSY-II subtests related to executive function, including Inhibition, Auditory Attention, Statue, and Design Fluency. Descriptive statistics indicated that children with comorbid ADHD and dyslexia generally exhibited lower scores across executive subtests compared to those with either ADHD or dyslexia alone. Mean scores and standard deviations for each measure are presented in Table 1.

Four separate one-way ANOVAs were conducted to compare group performance across diagnostic categories for each executive function measure (Inhibition, Auditory Attention, Statue, Design Fluency). Statistically significant differences emerged for Inhibition (*p* = 0.003) and Auditory Attention (*p* = 0.007), with children in the comorbid group performing more poorly on these tasks, suggesting greater executive function impairment in this subgroup. Table 2 presents the one-way ANOVA results for NEPSY-II executive function subtests by diagnostic group. Given small and unequal group sizes, ANOVA assumptions were considered cautiously; results are interpreted as exploratory.

η^2^ represents effect size (proportion of variance explained). Values of 0.01, 0.06, and 0.14 are typically interpreted as small, medium, and large effects, respectively. Despite statistically significant findings for several domains, results should be interpreted cautiously due to small and uneven group sizes.

Effect size estimates indicated moderate to large effects for Inhibition (η^2^ = 0.34), Auditory Attention (η^2^ = 0.31), and Design Fluency (η^2^ = 0.46), suggesting meaningful group differences despite the small sample sizes.

Given the unequal group sizes, exploratory post hoc comparisons (Games–Howell) were conducted to examine pairwise differences between diagnostic groups. These analyses suggested that the comorbid group tended to perform more poorly than the ADHD-only and dyslexia-only groups, particularly on inhibition and auditory attention tasks. However, these comparisons should be interpreted cautiously due to limited statistical power. Although visual overlap between boxplots was substantial, ANOVA evaluates differences in group means relative to within-group variance rather than complete distribution separation. Given the small and uneven group sizes, these findings were interpreted cautiously and used primarily to inform the subsequent qualitative profiling stage.

**Figure 1:**
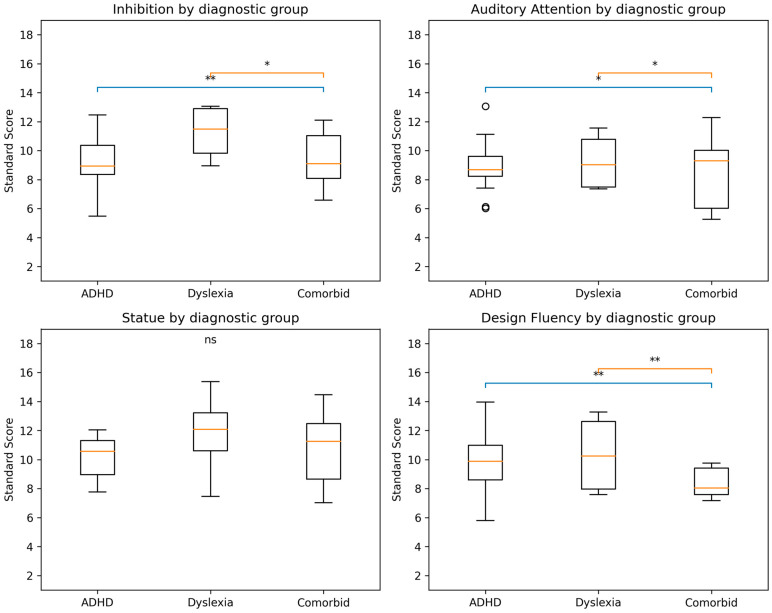
Standard scores on NEPSY-II subtests across ADHD, Dyslexia, and Comorbid diagnostic groups. Each subplot illustrates performance on one subtest: Inhibition, Auditory Attention, Statue, and Design Fluency. Boxes represent interquartile ranges, horizontal lines indicate medians, and dots represent outliers. Note. Exploratory post hoc comparisons (Games–Howell) are illustrated for descriptive purposes (* *p* < 0.05, ** *p* < 0.01). Considerable overlap between groups was observed across several subtests. No significant group differences were observed for the Statue subtest (ns). Given the small and unequal group sizes, all inferential findings should be interpreted cautiously and considered exploratory. Blue and orange lines indicate pairwise post hoc comparisons between diagnostic groups.

Boxplot visualizations highlighted the overlap and variability within groups and underscored the need for nuanced, clinically focused interpretation. Exploratory pairwise comparisons are visually represented in Figure 1; however, substantial overlap between groups was observed, reinforcing the need for cautious interpretation.

Figure 1 presents interquartile ranges, with medians and outliers across ADHD, Dyslexia, and Comorbid diagnostic groups, for the NEPSY II attention and executive functions subtests: auditory attention, inhibition, statue and design fluency.

These descriptive and visual analyses informed the development of the qualitative typology in Stage 2, providing a group-level context for understanding executive function variability in ADHD, dyslexia, and comorbid presentations. Rather than serving as confirmatory statistical evidence, the Stage 1 analyses were intended to identify broad patterns of executive-function variability across diagnostic presentations. The observed overlap and heterogeneity between groups highlighted the limitations of categorical comparisons and informed the subsequent qualitative profiling process. Specifically, domains showing recurrent variability in inhibition, auditory attention, working memory, and processing speed were explored further through cross-case thematic synthesis in Stage 2. Thus, the quantitative findings primarily functioned as an exploratory contextual framework for the development and interpretation of the cognitive executive profiles.

### 3.2. Stage 2: Clinical Typology of Executive Function Profiles (N = 11)

A qualitative synthesis was conducted based on 11 individual clinical evaluations of children aged 9 to 13 with ADHD (inattentive type), developmental dyslexia, or comorbid presentations. The analysis followed a semi-inductive thematic approach [17], allowing for the identification of recurrent executive functioning patterns across cases.

#### 3.2.1. Identification of Cognitive Executive Profiles

Through cross-case comparison and thematic coding of standardized test results (NEPSY-II, WISC-IV, and WJ-IV), three distinct cognitive executive profiles were identified:

1. *Globally reduced Executive (n = 4):* Children in this profile exhibited consistently low scores across NEPSY-II executive function subtests (Inhibition, Auditory Attention, Response Set, Design fluency), with additional deficits in working memory and processing speed (WISC-IV). Academic achievement scores (WJ-IV) indicated significant delays, particularly in reading fluency and phonological processing. This profile was predominantly associated with comorbid ADHD and dyslexia.
2. *Verbal–mnestic executive vulnerability (n = 4):* Characterized by relative strengths in visuomotor and design fluency tasks but marked weaknesses in auditory attention, narrative memory, and verbal working memory. WISC-IV profiles showed average to below-average verbal comprehension and working memory. Academic performance showed variable reading accuracy but reduced written expression and verbal recall. Most children in this group met criteria for developmental dyslexia without ADHD.
3. *Selective executive control deficit (n = 3):* This profile included children with relatively preserved performance in structured tasks but with selective deficits in inhibitory control and attentional regulation, particularly in less structured or cognitively demanding contexts. WISC-IV findings revealed average verbal comprehension with lower working memory. WJ-IV scores were generally within the average range. All children in this group had ADHD (inattentive type) without comorbid dyslexia.

Some cases showed partial overlap between profiles; in such instances, classification was based on the predominant pattern of executive functioning.

#### 3.2.2. Narrative Patterns and Clinical Profile Integration

**Figure 2:**
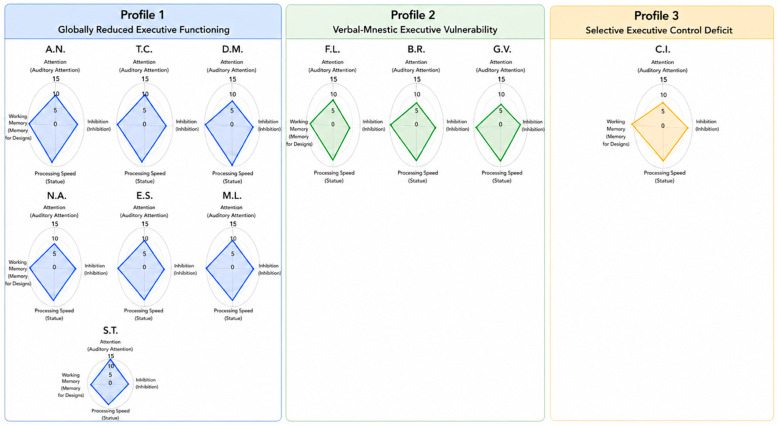
Executive Function Profiles by Case (Grouped by profile types). Note. Higher scores (closer to the outer ring) indicate poorer performance. The four domains correspond to NEPSY-II subtests: Attention (Auditory Attention), Inhibition, Working Memory (Memory for Designs), and Motor persistence (Statue). Case labels represent anonymized participant identifiers. Scores reflect standardized performance levels categorized as low (<8), medium (8–11), and high (>11).

Cross-case comparison supported the consistency of the three cognitive executive profiles across multiple neuropsychological domains. The globally reduced executive profile was characterized by broad impairments in inhibition, attention regulation, working memory, and academic functioning, most frequently observed in children with comorbid ADHD and dyslexia. In contrast, the verbal–mnestic executive vulnerability profile reflected more circumscribed language-mediated executive difficulties, particularly affecting verbal memory and phonological processing, despite relatively preserved reasoning abilities. The selective executive control deficit profile was associated with more focal difficulties in inhibition and self-regulation, especially in less structured contexts, while general cognitive functioning remained comparatively preserved. Table 3 and Figure 2 provide complementary visualization of these profile patterns across cases.

Clinical implications. The identification of these profiles enhances differential diagnosis and supports individualized intervention planning. These results highlight the heterogeneity of executive functioning deficits in neurodevelopmental disorders and underscore the clinical value of typological profiling derived from real-world psychological assessment data.

### 3.3. Integrated Executive Function Profiles

To illustrate the refined clinical typology derived from thematic synthesis, three executive functioning profiles are exemplified using selected individual cases. Each narrative highlights distinct patterns of strengths and vulnerabilities, consistent with the following clinical executive categories: globally reduced executive, verbal–mnestic executive vulnerability, and selective executive control deficit. Each vignette includes contextualized neurocognitive data and a summary interpretation.

*Profile 1: Globally reduced executive.* This profile is defined by diffuse impairments across multiple executive domains, including inhibition, attention control, working memory, and academic performance. Children in this group often present with comorbid ADHD and dyslexia and require intensive academic and behavioral support.

*Profile 2: Verbal–mnestic executive vulnerability.* Children in this group present with verbal and phonological memory difficulties that affect academic performance, despite preserved reasoning or verbal skills. These profiles are most frequently associated with developmental dyslexia.

*Profile 3: Selective executive control deficit.* This profile reflects more focal executive control issues—typically impulsivity, response inhibition, or processing speed—in children whose cognitive profiles are otherwise less impaired. These children may demonstrate high variability in behavior despite intact reasoning skills.

Figure 2 presents the 11 individual radar charts grouped into three distinct cognitive executive profiles: (1) globally reduced executive, (2) verbal–mnestic executive vulnerability, and (3) selective executive control deficit.

## 4. Discussions

The present study aimed to examine executive function heterogeneity in children with ADHD, developmental dyslexia, and comorbid presentations using a clinically grounded, mixed-method approach. Through the integration of standardized neuropsychological assessment data and case-based thematic synthesis, three distinct cognitive executive profiles were identified: globally reduced executive functioning, verbal–mnestic executive vulnerability, and selective executive control deficit. Importantly, these profiles suggest potential patterns that may extend beyond categorical diagnoses, although this interpretation remains preliminary given the exploratory nature of the study. This finding is consistent with emerging person-centered research suggesting that executive function configurations may capture clinically meaningful variability not always reflected by symptom-based classifications alone [10,11]. By deriving profiles directly from real-world clinical assessment data, the present study extends this line of research by demonstrating the feasibility and clinical relevance of executive function profiling in applied settings, where diagnostic complexity and intra-individual variability are the norm rather than the exception.

### 4.1. Stage 1

Although statistically significant differences were observed in selected NEPSY-II subtests (e.g., Inhibition, Auditory Attention), these findings should be interpreted cautiously given the small and uneven group sizes in Stage 1 (N = 30). The heterogeneity of diagnostic presentations and the particularly small size of the dyslexia-only group limit the robustness of statistical comparisons. Therefore, the quantitative findings were used primarily to inform the subsequent qualitative analysis rather than to support strong inferential conclusions.

As Kazdin [14] emphasizes, exploratory analyses based on clinical case data play a critical role in bridging research and practice. They allow for the identification of meaningful trends that can guide future hypothesis-driven studies while enhancing clinical decision-making through data-informed typologies. This aligns with recent developments in mixed-methods research, which highlight the value of integrating qualitative depth with quantitative structure to capture the complexity of psychological phenomena in applied contexts [13].

Mixed-method approaches have been especially relevant in clinical child psychology, where the interplay between idiographic and nomothetic data supports more nuanced understanding of diagnostic diversity and treatment needs.

Thus, even in the absence of significant group-level effects, the patterns observed offer valuable insights into the cognitive diversity present in neurodevelopmental profiles. These findings underscore the importance of methodological pluralism and clinically contextualized research in advancing child and adolescent mental health assessment.

### 4.2. Stage 2

Stage 2 of the present study employed a qualitative case-based thematic synthesis of 11 individual psychological evaluation reports to explore cognitive executive diversity in children diagnosed with ADHD, dyslexia, or comorbid presentations. Through a structured thematic analysis, three distinct cognitive executive profiles were identified: (1) globally reduced executive functioning, (2) verbal–mnestic executive vulnerability, and (3) selective executive control deficit. These profiles offer a clinically meaningful typology that complements categorical diagnostic labels by capturing additional intra-individual variability.

These profiles were not defined by formal diagnoses alone but emerged through synthesis of performance patterns across NEPSY-II, WISC-IV, and WJ-IV subtests. This supports the clinical value of profile-based assessment approaches, where patterns of strength and vulnerability are interpreted functionally, rather than strictly normatively. The clinical typology derived from this synthesis aligns with person-centered EF models that have demonstrated predictive validity for academic, behavioral, and emotional outcomes [10,11]. From a neurocognitive perspective, these profiles may reflect differential involvement of executive control networks, including frontostriatal and frontoparietal systems, which are known to support inhibition, working memory, and cognitive flexibility. Variability in these networks may contribute to the observed heterogeneity across clinical presentations.

Children in the *globally reduced executive functioning* profile exhibited low performance across nearly all executive domains, including inhibition, flexibility, auditory attention, and working memory. These children typically presented with comorbid ADHD and dyslexia and showed significant academic delays. This profile underscores the need for comprehensive, multimodal interventions targeting both cognitive regulation and literacy skills, as well as possible referrals for learning accommodations.

The *verbal–mnestic executive vulnerability* profile included children with relatively preserved performance in visuospatial and inhibition tasks but marked weaknesses in verbal memory, attention regulation, and planning. This group primarily consisted of children with dyslexia only, highlighting the cognitive strain involved in compensating for phonological deficits. Interventions for this group may benefit from leveraging visual strengths while explicitly supporting verbal working memory and organizational strategies.

The selective executive control deficit profile captured children with ADHD (predominantly inattentive type), who performed relatively well on structured tasks of memory and inhibition, yet demonstrated impulsivity and poor self-regulation in less structured or contextually ambiguous tasks (e.g., clocks, response set shifting). This pattern reflects the contextual variability often seen in ADHD and aligns with models of executive functioning that emphasize the role of motivation, time perception, and contextual demands [5].

Recent empirical research supports the clinical validity of this profiling approach. For instance, Cumming [15] identified EF profiles in young children that predicted risk for behavioral and emotional disorders. Their findings echo the current study’s themes of inhibition, working memory, and flexibility as central differentiators of cognitive emotional risk. Similarly, Wang et al. [16] demonstrated that executive function mediates the effects of parenting practices on internalizing and externalizing problems, reinforcing the importance of ecological influences in shaping EF development.

Becker et al. [10] further highlight the added value of EF profiles over categorical diagnoses, showing that person-centered executive function profiles are strong predictors of adolescent ADHD symptoms and academic performance. The current study’s results echo this model, suggesting profile patterns associated with distinct clinical support needs that may not be fully reflected by diagnostic categories alone.

### 4.3. Implications for Practice and Policy

Growing evidence underscores the translational potential of EF profiling. For example, van Stralen et al. [21] showed that pharmacological interventions such as extended-release guanfacine and psychostimulants have differential impacts on EF domains, highlighting the clinical relevance of personalized EF profiles in guiding treatment. The profiles identified in the current study likewise suggest that neurocognitive diversity necessitates tailored support rather than uniform intervention.

These conclusions are further supported by recent person-centered analyses. Skogli et al. [11] showed that data-driven neuropsychological profiling could identify ADHD-related subgroups with differing levels of academic and emotional risk—an approach paralleled by the qualitative synthesis presented here. Together, these studies argue for the routine use of individualized executive profiling in both diagnostic formulation and treatment planning.

From a clinical and educational perspective, the identified profiles may support more individualized intervention planning. Children within the globally reduced executive profile may benefit from multimodal interventions integrating executive function scaffolding, literacy remediation, and behavioral regulation support, alongside classroom accommodations such as reduced task load and structured instructional sequencing. In contrast, children with verbal–mnestic executive vulnerability may respond better to visually mediated instruction, explicit organizational supports, and reduced verbal working memory demands. For children with selective executive control deficits, interventions targeting inhibition, self-monitoring, and contextual behavioral regulation may be particularly relevant, especially in less structured academic environments.

### 4.4. Limitations and Future Directions

This study presents several limitations that should be acknowledged. First, the small sample size, particularly in Stage 2 (N = 11), restricts the generalizability of findings and precludes statistical inference. The aim of the present study was not statistical generalization but the development of clinically informative and transferable executive function profiles applicable to similar assessment contexts. While the case-based thematic synthesis was appropriate for exploring cognitive heterogeneity in clinical practice, future research should aim to replicate the identified executive function (EF) profiles using larger, more diverse samples and quantitative classification methods (e.g., cluster or latent profile analysis).

Second, the data were collected retrospectively from routine clinical assessments, which may have introduced variability in assessment conditions and case selection. Although all children were assessed using standardized instruments and a consistent protocol, the absence of control over referral criteria and environmental factors (e.g., school or family context) may have influenced individual performance. Including additional sources of information, such as teacher ratings or classroom observations, could enrich the ecological validity of the profiles and support a more comprehensive understanding of real-life executive functioning.

Third, the analytic framework was constrained by the available subtests and the structure of norm-referenced instruments, limiting the capacity to explore emotional and motivational dimensions of executive control. Instruments such as the Conners Rating Scales (Conners 3), Wisconsin Card Sorting Test (WCST), or the Children’s Stroop Test could complement the current protocol by capturing dynamic and context-sensitive aspects of cognitive flexibility, inhibition, and emotional regulation.

Fourth, the profiles developed in this study are cross-sectional and may not reflect the developmental trajectory of executive functioning over time. Longitudinal follow-up studies could explore the stability of these profiles and their predictive value for academic achievement, behavioral regulation, and socio-emotional outcomes. Additionally, future research could examine how environmental variables—such as parenting practices, school accommodations, and intervention responsiveness—interact with EF profiles to shape clinical trajectories.

Despite these limitations, the current study demonstrates the feasibility and clinical relevance of deriving executive function profiles from standardized psychological assessments. By integrating idiographic case data with structured thematic synthesis, this approach contributes to a growing body of mixed-methods research aimed at enhancing diagnostic formulation, intervention planning, and individualized care in developmental psychopathology.

## 5. Conclusions

This study contributes to the growing body of research emphasizing the value of individualized, clinically grounded approaches to understanding executive functioning in children with ADHD, dyslexia, and comorbid presentations. By integrating data from standardized assessments through thematic synthesis, the research identified three distinct cognitive executive profiles that transcend traditional diagnostic categories. These profiles—globally reduced executive functioning, verbal–mnestic executive vulnerability, and selective executive control deficit—highlight the heterogeneity of executive functioning difficulties in neurodevelopmental conditions. The findings support the clinical utility of profile-based interpretation and underscore the potential of mixed-methods designs in bridging standardized data with nuanced case conceptualization. Such approaches offer a pathway for more precise, context-sensitive, and personalized interventions in clinical child psychology.

## Figures and Tables

**Table 1:** NEPSY-II Descriptive Statistics by Diagnostic Group.

Group	*n*	Inhibition Mean	Inhibition SD	Auditory Attention Mean	Auditory Attention SD	Statue Mean	Statue SD	Design Fluency Mean	Design Fluency SD
ADHD	18	9.3	2.0	9.1	2.1	10.5	1.8	9.7	2.0
Dyslexia	4	10.8	2.2	10.0	2.0	11.3	1.9	10.5	2.4
Comorbid	8	8.2	2.5	8.0	2.3	9.8	2.2	8.9	2.1

Note. Means and standard deviations are reported for each NEPSY-II subtest by diagnostic group. *n* = sample size per group. ADHD = Attention Deficit Hyperactivity Disorder.

**Table 2:** One-Way ANOVA Results for NEPSY-II Executive Function Subtests by Diagnostic Group.

Domain	df1	df2	F	*p*	η^2^
Inhibition	2	27	7.05	0.003	0.34
Auditory Attention	2	27	6.09	0.007	0.31
Statue	2	27	0.77	0.475	0.05
Design Fluency	2	27	11.46	<0.001	0.46

Note. df1 = degrees of freedom between groups. df2 = degrees of freedom within groups. *p* values reflect comparisons across ADHD, dyslexia, and comorbid groups. Significant group differences were observed for Inhibition and Auditory Attention. However, given the exploratory design and sample characteristics, results were interpreted primarily in a descriptive and hypothesis-generating framework.

**Table 3:** Clinical cognitive profile matrix (N = 11).

Case	Diagnosis	NEPSY-II Key Scores	WISC-IV Profile	WJ-IV Weaknesses	Emergent Theme	Clinical-Cognitive Profile
A.N.	ADHD	Low inhibition, poor memory	WMI = 80, PSI = 79	Very poor reading–writing	Executive deficits + slow processing	Profile 1—globally reduced executive
F.L.	Dyslexia	Weak auditory attention, medium inhibition	VCI = 118, PSI = 85	Poor phonological reading	Verbally compensated dyslexia	Profile 2—verbal–mnestic executive vulnerability
T.C.	ADHD + Dyslexia	Poor verbal memory, low inhibition	WMI = 85, VCI = 98	Slow, incoherent writing	Mixed attention and language deficits	Profile 1—globally reduced executive
D.M.	ADHD	Poor response control, slow motor speed	PRI = 92, PSI = 77	Slow writing, poor planning	Good visual processing but poor control	Profile 1—globally reduced executive
B.R.	Dyslexia	Low auditory memory	VCI = 108, WMI = 82	Below-level writing	Phonological memory deficits	Profile 2—verbal–mnestic executive vulnerability
N.A.	ADHD	Poor inhibition and attention	WMI = 84, PSI = 72	Disorganized writing	Globally reduced executive profile	Profile 1—globally reduced executive
C.I.	ADHD + Dyslexia	Medium working memory, poor attention	WMI = 86, PSI = 91	Reading–writing difficulties	Good execution but unstable attention	Profile 3—selective executive control deficit
E.S.	ADHD	OK inhibition, low processing speed	PSI = 76, VCI = 96	Slow writing	Slow processing without severe dysfunction	Profile 1—globally reduced executive
G.V.	Dyslexia	Medium visuomotor, poor memory	VCI = 110, PRI = 90	Incoherent reading	Good verbal logic but school difficulties	Profile 2—verbal–mnestic executive vulnerability
M.L.	Comorbid	Low inhibition, poor multitasking	WMI = 78, PSI = 68	Weak reading, writing, math	Globally impaired profile	Profile 1—globally reduced executive
S.T.	ADHD	Poor attention and inhibition	WMI = 83, PSI = 70	Disorganized writing	General executive deficits	Profile 1—globally reduced executive

Note. WISC-IV indexes’ scores: VCI = Verbal Comprehension Index; PRI = Perceptual Reasoning Index; WMI = Working Memory Index; PSI = Processing Speed Index. WJ-IV academic domains reflect age-equivalent weaknesses. NEPSY-II scores are qualitatively described and derived from Romanian norms.

## Data Availability

The original contributions presented in this study are included in the article. Further inquiries can be directed to the corresponding author.
